# Observations of dynamical behavior in a stochastic Wilson-Cowan population with plasticity

**DOI:** 10.1186/1471-2202-14-S1-P400

**Published:** 2013-07-08

**Authors:** Jeremy Neuman, Bert Kiewiet, Jack D Cowan, Wim van Drongelen

**Affiliations:** 1 Dept. of Physics, University of Chicago, Chicago, IL 60637, USA; 2 Dept. of Applied Mathematics, University of Twente, 7500 AE Enschede, Netherlands; 3 Dept. of Mathematics, University of Chicago, Chicago, IL 60637, USA; 4 Dept. of Pediatrics, University of Chicago, Chicago, IL 60637, USA

## 

Understanding network connectivity and its role in brain activity is an arduous task. Complicating matters further is the introduction of synaptic plasticity rules. Observations using a mean-field perspective [1] are by their nature incomplete so, here, a stochastic model, which includes fluctuations, has been employed. This analysis shows that two types of network connections, driven by plasticity, exhibit oscillatory behavior signaled by a flipping between Up and Down states. Fluctuations in each state in both setups display power law-like avalanche distributions.

This study, employing a stochastic algorithm [2] used previously in a population-based model [3], introduces plasticity, according to a modified version of [4], into both an E → E and I → E network (Figure 1A). The former network includes plastic excitatory, anti-Hebbian synapses, connecting the populations, while the latter contains plastic inhibitory Hebbian synapses. Both networks incorporate a constant recurrent excitatory synapse. Dynamically, each network undergoes oscillations of relaxation type (Figure 1B) with fluctuations whose avalanche distributions look like power laws (Figure 1C).

**Figure 1:**
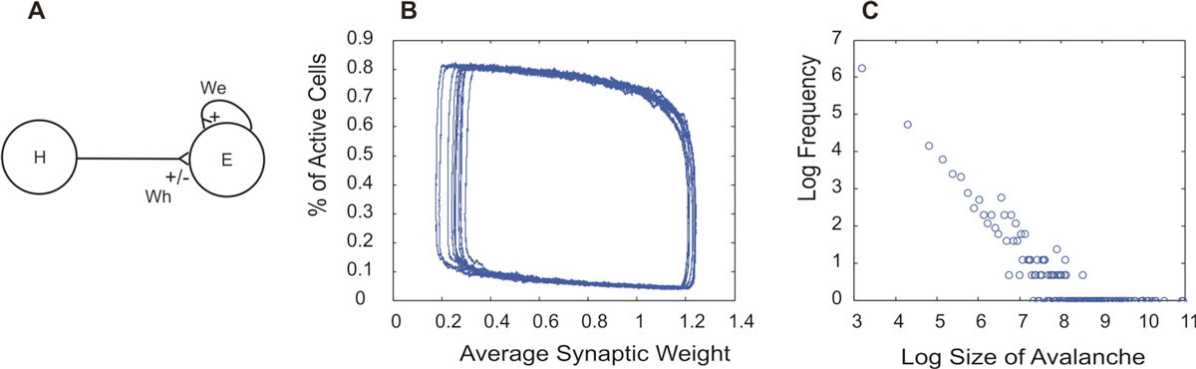
**Network configuration with two populations**. (A) Diagram of the connection. If H is an excitatory population, synapse Wh has anti-Hebbian plasticity. If H represents an inhibitory population, the synapse has Hebbian plasticity. (B) Phase plot of activity of E versus the strength of Wh in the scenario where H is an inhibitory network. (C) The avalanche distribution of the Up state in panel (B).

## Conclusions

Understanding the dynamics of plasticity-driven neural networks is vital. Here, it was shown that a stochastic Wilson-Cowan population connected to an exterior population can naturally exhibit relaxation oscillations. This result with its power law avalanche statistics is a potential sign of self-organized criticality.
